# Deciphering glial scar after spinal cord injury

**DOI:** 10.1093/burnst/tkab035

**Published:** 2021-11-08

**Authors:** Yu Zhang, Shuhai Yang, Chang Liu, Xiaoxiao Han, Xiaosong Gu, Songlin Zhou

**Affiliations:** Jiangsu Province Hospital of Chinese Medicine, Nanjing, 210000, China; Medical College of Nantong University, Nantong, 226001, China; Key Laboratory of Neuroregeneration of Jiangsu and Ministry of Education, NMPA Key Laboratory for Research and Evaluation of Tissue Engineering Technology Products, Jiangsu Clinical Medicine Center of Tissue Engineering and Nerve Injury Repair, Co-Innovation Center of Neuroregeneration, Nantong University, Nantong, Jiangsu Province, China; Key Laboratory of Neuroregeneration of Jiangsu and Ministry of Education, NMPA Key Laboratory for Research and Evaluation of Tissue Engineering Technology Products, Jiangsu Clinical Medicine Center of Tissue Engineering and Nerve Injury Repair, Co-Innovation Center of Neuroregeneration, Nantong University, Nantong, Jiangsu Province, China; Key Laboratory of Neuroregeneration of Jiangsu and Ministry of Education, NMPA Key Laboratory for Research and Evaluation of Tissue Engineering Technology Products, Jiangsu Clinical Medicine Center of Tissue Engineering and Nerve Injury Repair, Co-Innovation Center of Neuroregeneration, Nantong University, Nantong, Jiangsu Province, China; Key Laboratory of Neuroregeneration of Jiangsu and Ministry of Education, NMPA Key Laboratory for Research and Evaluation of Tissue Engineering Technology Products, Jiangsu Clinical Medicine Center of Tissue Engineering and Nerve Injury Repair, Co-Innovation Center of Neuroregeneration, Nantong University, Nantong, Jiangsu Province, China

**Keywords:** Spinal cord injury, Glial scar, Axon regeneration, Therapeutic strategy

## Abstract

Spinal cord injury (SCI) often leads to permanent disability, which is mainly caused by the loss of functional recovery. In this review, we aimed to investigate why the healing process is interrupted. One of the reasons for this interruption is the formation of a glial scar around the severely damaged tissue, which is usually covered by reactive glia, macrophages and fibroblasts. Aiming to clarify this issue, we summarize the latest research findings pertaining to scar formation, tissue repair, and the divergent roles of blood-derived monocytes/macrophages, ependymal cells, fibroblasts, microglia, oligodendrocyte progenitor cells (OPCs), neuron-glial antigen 2 (NG2) and astrocytes during the process of scar formation, and further analyse the contribution of these cells to scar formation. In addition, we recapitulate the development of therapeutic treatments targeting glial scar components. Altogether, we aim to present a comprehensive decoding of the glial scar and explore potential therapeutic strategies for improving functional recovery after SCI.

Highlights

- Illustration of scar formation by different cell types around the damaged area.
- Different roles that glia plays in different stages of scar formation.
- Multiple ways of therapy for promoting axon regeneration after SCI.

## Background

The incidence of spinal cord injury (SCI) has increased in recent years. According to the World Health Organization, an estimated 250,000–500,000 people suffer from SCI each year. Owing to the limited self-regenerative ability of the central nervous system (CNS), post-SCI neurological deficits are often permanent, depending on the site of injury and severity [1]. Disability caused by SCI often leads to loss of employment and imposes a heavy burden on affected families and society. Therefore, the development of therapeutic strategies to improve post-SCI functional recovery is a key research imperative [2].

The formation and impact of glial scars have been studied thoroughly in SCI, and glial scars are formed after cortical injury, ischemic brain injury or neuron inflammation. Glial scars mainly consist of two parts: a border line and a lesion core. After undergoing CNS damage, three primary cell types: activated astrocytes, newly proliferated microglia and oligodendrocyte progenitor cells (OPCs), which are a type of neuroglia identified by the expression of neuron-glial antigen 2 (NG2), also known as chondroitin sulfate proteoglycan 4 (CSPG4), and platelet-derived growth factor receptor-α (PDGFRα) surround the damaged center (lesion core), forming a dense border line. While the lesion core contains few glial cells, the core is made up of a large amount of extracellular matrix proteins (ECM) that show high inhibition of axonal growth and regeneration, vascular-derived fibroblasts, ependymal cells and blood-derived monocytes/macrophages.

The pathogenesis of neurological deficits following SCI involves a complex interaction between astrocytes, microglia, oligodendrocytes, immune cells and vascular systems [3,4]. In post-SCI imaging of the damaged area, a cystic cavity is formed due to bleeding, necrosis, exudation and liquefaction of necrotic substances filled by astrocytes and microglia. The cystic cavity is a part of the scar and tends to be stable as the injury progresses. Currently, there is no clear consensus on the role of various constituents of glial scars in axon regeneration. Therefore, elucidation of the causes of glial scar formation and identification of the cell components involved and the related regulatory mechanisms may help regulate post-SCI scar formation and promote axon regeneration.

## Review

### Causes of glial scar fornation

**Figure 1.**
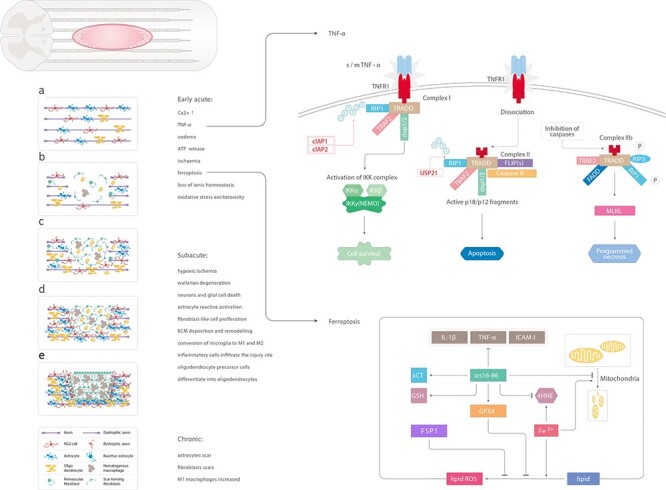
Schematic illustration of the main changes induced by spinal cord injury. (**a**) Uninjured spinal cord. (**b**) Early acute phase of spinal cord injury: increased level of Ca^2+^, TNF-α, edema, ATP release, ischemia, ferroptosis, loss of ionic homeostasis, oxidative stress excitotoxicity. Soluble TNF-α binds to TNFR1 on the cell membrane. RIP1 binds to other related proteins to form complex 1. cIAP1 and cIAP2 can ubiquitinate RIP1 and activate the NF-κB signaling pathway to promote cell survival. USP21 induces deubiquitination of RIP1 to form complex 2, leading to apoptosis. Fe^2+^ catalyzes the peroxidation of liposomes on the cell membrane and increases the production of reactive oxygen species. In addition, GPX4 is inactivated, thereby inducing iron-dependent death of cells. The iron-dependent death inhibitor, SRS 16–86, significantly inhibits the expression of inflammatory factors IL-1β, TNF-α and ICAM-1 and rescues the reduction of mitochondria and crest reduction. (**c**, **d**) Subacute phase: hypoxic ischemia, reactive astrocytosis, Wallarian degeneration, neurons and glial cell death, reactive activation of astrocytes, fibroblast-like cell proliferation, ECM deposition and remodeling, conversion of microglia to M1 and M2, infiltration of inflammatory cells at the injury site, differentiation of OPCs into oligodendrocytes. (**e**) Chronic phase: formation of astrocyte scars, fibroblast scars and increase in M1 macrophages. *TNF- α* tumor necrosis factor-alpha, *ATP* adenosine triphosphate, *RIP1* receptor interacting protein 1, *cIAP1* apoptosis inhibitory protein 1, *GPX4* glutathione peroxidase 4, *IL-1β* interleukin-1β, *ICAM-1* intercellular adhesion molecule-1, *OPCs* oligodendrocyte progenitor cells

Inhibition of neuronal and glial cell death after SCI is a fundamental approach for reducing glial scars. Promoting cell survival helps preserve the anatomical integrity of the spinal cord, which is essential for post-SCI functional recovery [5]. The pathophysiology of SCI involves complex molecular and cellular biological processes, which can be divided into two stages: primary injury and secondary injury [6]. Primary injury refers to the immediate effect of violence on the spinal cord, including contusion, partial or complete transection of the spinal cord, and associated bleeding and edema. Secondary injury refers to post-traumatic ischemic injury to several spinal cord segments caused by vasospasm. In addition, disruption of the blood–brain barrier leads to infiltration of inflammatory cells into the injury site. The degradation of damaged axons, demyelination of axons and release of toxic myelin disintegration products lead to the death of a large number of neurons and ~50% of oligodendrocytes and astrocytes in the injured area [7]. Liquefactive necrosis results in the formation of cavities of varying sizes. Subsequently, the proliferation of the surrounding glia and fibrous tissues, formation of thick arachnoid adhesions and eventual formation of cysts occurs, which further leads to dysfunction (Figure 1).

Primary injury can lead to immediate cell death; however, appropriate therapeutic interventions can help prevent cell death due to secondary injury [8]. Necrosis, apoptosis and necroptosis are the predominant modes of cell death after SCI [9,10]. Autophagy has a protective effect against cell damage caused by SCI [11]. Ferroptosis is a newly discovered iron-dependent mode of programmed cell death that is different from apoptosis, necrosis and autophagy [12]. Ferroptotic cell death is characterized by reduced cell mitochondrial volume, increased mitochondrial membrane density and a reduced number of cristae [13]. Execution of ferroptosis is known as an iron-catalyzed excessive peroxidation of polyunsaturated fatty acid (PUFA)-containing phospholipids (PLs), which occurs in excess in mammalian cell membranes [14,15]. Moreover, glutathione peroxidase 4 (GPX4) is inactivated, which induces ferroptotic cell death. Zhang *et al*. [16] demonstrated the involvement of ferroptosis in post-SCI neuronal death in a rat model of SCI; on electron microscopy, the mitochondria were found to exhibit characteristics of ferroptosis. SRS 16–86, a third-generation highly selective ferroptotic inhibitor, was shown to significantly improve the Basso, Beattie and Bresnahan locomotor scores for hind limb function after SCI, and significantly inhibit the expression of inflammatory factors interleukin-1β (IL-1β), tumor necrosis factor-α (TNF-α) and intercellular adhesion molecule-1(ICAM-1) [16]. This may be related to post-SCI bleeding and elevated concentrations of local ROS and the excitatory neurotransmitter glutamate [17,18] (Figure 1b).

TNF-α is an inflammatory cytokine that induces cell survival, apoptosis and necrotic apoptosis after SCI. After SCI, TNF-α is produced by activated macrophages and monocytes in the surrounding damaged tissue and blood, and free TNF-α binds to tumor necrosis factor receptor 1 (TNFR1) on the cell membrane. Complex I is then formed by recruitment of the TNF receptor-related death domain (TRADD) and receptor-interacting protein 1 (RIP1) [19], TNF receptor-related factor 2 (TRAF2), apoptosis inhibitory protein 1 (cIAP1) and cIAP2. The formation of complex I is a common pathway for cell survival, apoptosis and necrotic apoptosis. At this stage, the ubiquitination state of RIP1 determines cell fate [20]. If RIP1 is ubiquitinated, it activates the nuclear factor-κB (NF-κB) signaling pathway while inhibiting apoptosis and necrotic apoptosis pathways to promote cell survival. Deubiquitinated RIP1 causes blockage of the NF-κB pathway, and subsequently, RIP3, TRADD, Fas death domain-related protein and caspase-8 form complex II, which promotes cell death [21]. Further exploration of the TNF-α signaling pathway after SCI, especially clarification of the potential involvement of other molecules in the regulation of RIP1 ubiquitination, may help promote the survival of neurons and glial cells, reduce the area of necrosis and promote post-SCI functional recovery (Figure 1a).

### Cell components involved in the formation of glial scars

As early as 1999, Prof. Silver’s team at the Case Western Reserve University documented the involvement of blood vessels and perivascular stromal cells, microglial cells, oligodendrocytes, OPCs, meningeal cells, reactive astrocytes, fibroblasts and blood-derived monocytes/macrophages in the formation of glial scars [22].

#### Microglial cells

Microglia can be classified into two types. One type is the resting state, which is essential for CNS environmental homeostasis. Another type is the reactive state induced by CNS injury, which can be further classified into two phenotypes (M1 and M2). Microglial cells are generally in a quiescent state; however, these cells can be activated by various stimuli [23]. Interferon-γ and LPS-TLR4 can induce the switch of microglial cells to the M1 type, which secrete TNF-α, IL-1β, IL-6 and IL-12; the proinflammatory and neurotoxic effects of these cytokines further aggravate SCI. IL-4, IL-13, IL-10 and TLRs can induce microglial cells to switch from M1 to M2 type, which secrete IL-10 and IL-13 and participate in the anti-inflammatory response and removal of cell debris [24]. In brief, the M1 phenotype exacerbates neuroinflammation, whereas the M2 phenotype promotes tissue repair and exerts anti-inflammatory effects. After SCI, microglial cells are initially transformed into the M1 and M2 types. Unlike other tissues that have self-generative abilities, M2-type microglial cells do not persist for a long time after SCI, and the M1 type eventually predominates over the M2 type [25]. The development of glial scars and the failure of CNS regeneration are probably related to a failed switch from M1 to M2 type. Therefore, how to propel microglial differentiation toward the M2 phenotype should attract interest as a potential therapeutic strategy.

As mentioned above, this probably oversimplifies the various states and different functions of microglia, specifically for example microglia roots in primitive yolk sac progenitors during embryogenesis [26] which can maintain self-renewal in adulthood [27]. The roles of microglia remain unclear because blood-derived monocytes rapidly infiltrate into the damaged tissue after SCI, and they can differentiate into macrophages and express many markers of microglia, which causes difficulties in discriminating between macrophages and microglia.

Recently, some efficient prediction tools, such as conditional gene targeting, have been developed, which have allowed the study of the specific roles of microglia. For example, in the Cx3cr1creER mouse line, microglia can be labeled with tamoxifen, while excluding monocyte-derived macrophages [28]. PLX5622, a colony-stimulating factor 1 receptor (CSF1R) inhibitor that crosses the blood–spinal cord barrier, can effectively deplete microglia [29]. With these deletion strategies, it has been demonstrated that microglia may have different roles depending on the context. In a stroke model, microglia can protect neurons by maintaining calcium levels. In contrast, it has been demonstrated that the deletion of microglia inhibited disease progression in Alzheimer’s disease models [30].

To explore the function of microglia after SCI, Lacroix and coworkers took advantage of Cx3cr1creER mice and found that microglia are an important component of the protective scar that forms after SCI. Microglia form a dense cellular interface between reactive astrocytes and infiltrating monocyte-derived macrophages at the border of the lesion post-SCI. After microglia depletion by PLX5622, the organization of the astrocytic scar was disrupted, the number of neurons and oligodendrocytes at the site of injury decreased and functional recovery was impaired [31].

Recently, Li *et al*. found that a crush injury to the spinal cord in neonatal mice presented scar-free healing, and the long projecting axons crossed the lesion. They demonstrated that microglia play critical roles in the nearly complete recovery of neonatal mice after SCI. Depletion of microglia in neonatal mice by Cx3cr1cre or PLX3397 significantly disrupted this healing process and inhibited axon regrowth. Specifically, they found that neonatal microglia could secrete fibronectin to form bridges in the ECM that connect the severed ends of the spinal cord and express peptidase inhibitors involved in resolving inflammation. Furthermore, they transplanted either neonatal microglia or adult microglia treated with peptidase inhibitors into spinal cord lesions of adult mice and found that these microglia significantly enhanced wound healing and axon regrowth [32].

Therefore, we suggest that transplantation of M2 into an injured site may be a potential strategy to provide a better microenvironment for axon regeneration. Interestingly, Kojima’s team conducted such an experiment [33]. M1 microglia incubated with GM-CSF (40 ng/mL) and M2 microglia incubated with IL-4 (40 ng/mL) were separately mixed with Matrigel and administered to the injured spinal cord site in an SCI mouse model. The results showed that compared with the control and M1 groups, the M2 microglia transplantation group showed significant recovery of motor function. At the same time, the transcriptional levels of some molecules that protect nerves, such as mannose receptor type C1 (Mrc1), arginase 1 (arg1) and insulin-like growth factor-1(IGF-1), increased significantly in the M2 microglia transplantation group. We predict that M2 could be a potential future target for the treatment of SCI.

Further technical progress in protein purification protocols, fluorescence-activated cell sorting (FACS), single-cell analysis, single-cell sequencing, translating ribosome affinity purification sequencing and unique markers will provide a deeper understanding of microglial heterogeneity and its effects on axon regeneration.

#### OPCs

**Figure 2.**
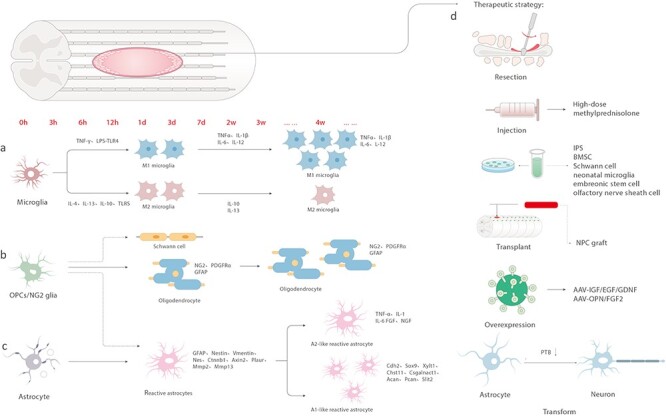
Temporal changes and treatment in spinal cord injury. (**a**) IFN-γ and LPS-TLR4 induce microglia to switch to M1 type within 3 days of injury and secrete TNF-α, IL-1β, IL-6 and IL-12. IL-4, IL-13, IL-10 and TLRs stimulate the switch to M2 type, which secrete IL-10 and IL-13. The M1 type eventually dominates. (**b**) OPCs/NG2 can differentiate into Schwann cells and astrocytes in the early stage of injury, or these can differentiate into oligodendrocytes and express NG2, PDGFRα and GFAP. (**c**) Astrocytes transform into reactive astrocytes after injury and up-regulate the expression of GFAP, nestin, vimentin, Nes, Ctnnb1, Axin2, Plaur, Mmp2 and Mmp13; however, over 2–4 weeks, A1-like astrocytes appear and up-regulate Cdh2, Sox9, Xylt1, Chst11, Csgalnact1, Acan, Pcan, Slit2 and another type of scar that secretes TNF-α, IL-1, IL-6, FGF and NGF. (**d**) Therapeutic strategy: surgical treatment, high-dose methylprednisolone, cell (iPS, BMSC, Schwann cell, neonatal microglia, embryonic stem cells, olfactory nerve sheath cells) transplantation, cocktail therapy, transforming astrocytes into neurons. *IFN-γ* interferon-gamma, *TNF- α* tumor necrosis factor-alpha, *IL* interleukin, *OPCs* oligodendrocyte progenitor cells, *GFAP* glial fibrillary acidic protein, *FGF* fibroblast growth factor, *NGF* nerve growth factor

OPCs, also known as NG2 expressing cells, which can proliferate at the edge of glial scars, are significantly activated after SCI. OPCs can differentiate into oligodendrocytes, which contribute to remyelination, hypertrophy and increased expression of NG2 [34]. NG2 is a CSPG that may mediate crosstalk between neurons and glial cells. In particular, NG2 (gene name cspg4) inhibits axon growth [35,36]. However, the overlap of its marker proteins with other cells masks the actual role of OPCs in the context of post-SCI repair. After SCI, NG2 expression is upregulated at the damage site, but macrophages, pericytes and non-myelinating Schwann cells also express NG2 [37]. In addition to expressing NG2 and platelet-derived growth factor receptor-α (PDGFRα), OPCs can also express glial fibrillary acidic protein (GFAP), an astrocyte marker protein, and can even differentiate into astrocytes. Many studies have found that Wnt, fibroblast growth factor (FGF) and PDGFR are involved in NG2 glial proliferation and migration to the injury site. However, these migrated NG2 expressing glial cells differentiate into astrocytes and Schwann cells, rather than myelinating oligodendrocytes (Figure 2) [4]. Thus, OPC functions are still controversial to some extent because of the common upregulation of proteoglycan NG2 in divergent cell types in the scar after injury and less differentiation into myelinating oligodendrocytes.

To further clarify the role of OPCs in the formation of post-SCI glial scars, the NG2-Cre estrogen receptor (CreER) strain, Olig2-CreER and PDGFRα-CreER strains, using genetic lineage tracing technology combined with molecular markers, can be used. To better understand this technology, we consider NG2-CreER as an example. These NG2-CreER mouse lines express the tamoxifen-inducible Cre recombinase under the control of the mouse NG2 (CSPG4) promoter. When mice containing loxP-flanked sequences are bred to the NG2-CreER mice, tamoxifen-inducible Cre-mediated recombination is expected to result in deletion of the floxed sequences in the Cre recombinase-expressing tissues of later generations. These NG2-CreER mice may be useful for inducible Cre recombinase expression in NG2-expressing glia, such as OPCs, which could express CSPG4 in the CNS.

However, each mouse line has certain disadvantages. The NG2-CreER strain has a low recombination efficiency of ~30–40% in NG2 cells [38] and pericytes [39]. The PDGFRα-CreER strain has a high recombination efficiency and labels fibroblasts [37]. Olig2-CreER mice label oligodendrocytes and astrocytes [40]. In a mouse model of optic nerve injury at the supraoptic colliculus with PTEN and SOCS3 co-knockout or co-expression of OPN/IGF1/CNTF, the regenerated axons were found to form functional synaptic connections with target organs; however, there was no significant functional recovery because the regenerated axons were not wrapped in myelin [41]. Further investigations are warranted to determine whether OPCs can be manipulated to remyelinate axons that cross the glial scar area. Generally speaking, OPCs engage in the formation and dissolution of glial scars rather than simply serving as a source for generating oligodendrocytes.

#### Astrocytes

SCI induces reactive activation of astrocytes, followed by transient changes in gene expression, cellular hypertrophy, migration and proliferation. The degree of activation is related to the distribution of proinflammatory cytokines and chemokine receptors on the cell surface. After the transfer of reactive astrocytes from the injured spinal cord to the normal spinal cord, the cells exhibited a dynamic switch from the reactive to the resting phenotype [42]. Reactive astrocytes were hypertrophic and densely distributed at the edges of the injured area. These cells show high expression of intermediate silk proteins such as GFAP, nestin and vimentin, and the cells migrate and eventually form a barrier-like structure.

Transcriptional profiling of reactive astrocytes from ischemic brain injury and neuroinflammation mouse models indicated that although a small group of genes is shared, reactive astrocytes upregulate genes specific to the type of injury or disease, which can be grouped into two types: A1 and A2. Liddelow *et al*. identified the functional characteristics of neuroinflammation-induced reactive astrocytes (A1) with the secretion of neurotoxins, which promotes neuronal cell and oligodendrocyte death. Surprisingly, they also characterized the same kind of reactive astrocytes, A1, in patients with neurodegenerative disease, which may suggest a new cell-target that could be investigated for therapy. However, [43] presented a contrasting result that the A2 type of reactive astrocytes induced by ischemic stroke may acquire a more protective phenotype with high expression of neurotrophic factors and transfer mitochondria to injured neurons. The mechanism leading to disparate outcomes remains unclear, but evidence suggests that the environmental milieu may greatly affect the response of astrocytes.

The proliferation of astrocytes depends on the STAT-3 signaling pathway and leucine zipper kinase (LZK, MAP3K13). Some reactive astrocytes are derived from astrocytes of ependymal origin; however, these cells are relatively few [44]. Astrocytes are tightly interconnected and continually reshape the boundary of the injury site, encapsulate immune cells and fibroblast-like cells through ephrin-mediated cell adhesion, and spatially isolate the residual nerve tissue from the damaged and fibrotic tissues. Astrocytes are the main cellular component of glial scars and play a key role in post-SCI pathological processes, including inflammation and tissue and ECM reconstruction. Astrocytes, together with other cells and extracellular factors, form subacute and chronic glial scar cells and extracellular microenvironments.

In addition, astrocytes secrete inflammatory cell chemokines, and, surprisingly, their toll-like receptors (TLRs) respond to infection by the innate immune system, leading to the investigation of the relationship between astrocytes and microglia, which can engage in phagocytosis and are involved in immune responses. Elkabes and coworkers of New Jersey State University studied the effect of TLR9 on the chemokines released by astrocytes, the polarization of macrophages, and the repair of SCI in mice through *in vitro* co-culture and mouse spinal cord contusion models [45]. The research team co-cultured spinal cord astrocytes and peritoneal macrophages in the Transwell system to detect the chemotaxis and polarization of astrocytes and their conditioned medium (CM) on macrophages. The results showed that compared with the control group, astrocytes treated with ODN 2088, an antagonist of TLR9, significantly increased the chemotaxis of macrophages by releasing chemokine (C-C motif) ligand 1 (CCL1) and further promoted their polarization to the M2 type. In addition, after astrocytes were treated with ODN 2088, the release of CCL2 and ccl9 decreased, which promoted the acquisition of the M2 phenotype, indicating that CCL2 and ccl9 are negative regulators of M2 polarization. Because of the roles of microglia mentioned above, M2 can be regulated by the secretion of astrocytes. Astrocytes may present an indirect therapeutic strategy for axon regeneration and locomotor recovery.

To deepen the understanding of reactive astrocytes, some questions pertaining to reactive astrocytes arise. What is the response of astrocytes in specific subgroups to different types of CNS injuries? What factors (extrinsic or intrinsic) initially determine the formation of astrocyte heterogeneity?

#### Blood-derived monocytes/macrophages

Blood-derived monocytes/macrophages are an important part of glial scars. Circulating monocytes are blood mononuclear phagocytes that may differentiate into macrophages or dendritic cells after infiltrating the damaged CNS. Monocytes migrate to the injured site and differentiate into macrophages in a multiphasic manner, which has a variety of functions in the process of wound healing [24]. Mouse monocytes can generally be divided into phagocytic and pro-inflammatory Ly6C^hi^ and anti-inflammatory Ly6C^lo^ subtypes. Ly6C^hi^ monocytes are also described as Cx3Cr1^lo^ and CCR2^hi^, and Ly6C^lo^ monocytes are referred to as Cx3Cr1^hi^ and CCR2^lo^ [46]. Previous studies on SCI in Cx3Cr1^GFP^ mice showed that both Cx3Cr1^lo^ and Cx3Cr1^hi^ macrophages existed at the injury site, indicating that after SCI, both pro-inflammatory Ly6C^hi^ and anti-inflammatory Ly6C^lo^ monocytes contribute to the formation of macrophage populations [47].

Both microglia and macrophages exist in the injured spinal cord, but due to their similarity in phenotype and antigen, it is difficult to distinguish between the two groups. There are experiments using multiple chimeric models that have been used to solve this problem and prove that macrophages occupy the glial scar area in and around the fibrotic lesion center, while microglia almost only exist in the glial scar area [48,49]. Macrophages are divided into M1-like and M2-like macrophages. As previous experiments have shown that M1-like macrophages can kill nearby cells and prevent cell proliferation, while M2-like macrophages can promote cell proliferation and tissue growth [50], the following hypothesis was proposed: promoting M2-like polarization is beneficial in CNS injury (such as SCI) and cell regeneration is limited in CNS injury. The M1/M2 macrophages in the injured spinal cord were first studied by Kigerl *et al*. [25]. The study reported that, except for a transient increase in M2 macrophages on the seventh day, the SCI site was mainly composed of M1 macrophages. In addition, the authors used *in vitro* bone marrow-derived macrophage (BMDM) experiments to show that M2 macrophage-conditioned medium can enhance the growth of neurites even on inhibitory substrates. The persistence of M1 macrophages after SCI is in contrast to the typical wound healing process, which is mainly related to M2 macrophages [51]. Therefore, this increases the possibility that M1 macrophages persist after SCI, resulting in a chronic inflammatory state that hinders cell regeneration.

After SCI, the injured site immediately recruits surrounding neutrophils, which easily pass through the mechanically injured blood–brain barrier to enter the CNS, starting from the first hour after the injury and reaching a peak in the 24 hours after the injury [52]. In fact, studies have shown that after peripheral nerve injury, the removal of distal nerve stumps during Waller’s degeneration is mainly carried out by neutrophils rather than macrophages [53]. After neutrophils begin to undergo apoptosis, monocytes are recruited through chemoattractive signals, such as MCP-1 (Ccl2). Once at the injury site, monocytes differentiate into macrophages in response to the cytokines and chemokines present in the injury environment. Immune activation through mild inflammation promotes cell regeneration. This phenomenon has been confirmed in subsequent studies. After SCI, compared with training alone, systemic injection of lipopolysaccharide combined with rehabilitation training can improve the forelimb function of rats [54].

#### Ependymal cells

Ependymal cells of the adult mammalian spinal cord are well-characterized neural stem cells that are derived from ectodermal cells and can differentiate into neurons, astrocytes or oligodendrocytes. After SCI, the ependymal cells are activated, exhibit stem/progenitor cell properties and generate scar-forming astrocytes [55,56]. Minimal SCI can induce an endogenous ependymal cell response where ependymal cells proliferate and migrate, differentiating primarily into astrocytes. These ependymal cells in the ependyma or surrounding gray matter do not undergo apoptosis in the minimal SCI model. They show significant proliferation and migration at the level of the needle track at three days following SCI. Through tracking of DiI, GFAP^+^ ependymal cells were found to be within 70 μm of the region of the central canal at 14 days post-SCI [57]. Another study found that the post-SCI environment and the age of stem cells affect the final differentiation fate of ependymal cells. *In vivo*, juvenile ependymal cells, which are ependymal cells from postnatal day 21, tend to form glial scars after severe SCI. In contrast, cultures of juvenile ependymal cells *in vitro* generate more neurospheres and oligodendrocytes than adult cells [58].

Although these ependymal cells migrate rapidly to the site of injury after SCI and mainly differentiate into astrocytes *in vivo*, they can differentiate into neurons under appropriate conditions *in vitro*. In particular, M2 macrophages upregulate the expression of sirtuin 2 (SIRT2) in ependymal cells through the BDNF/TrkB-MEK/ERK signaling pathway. SIRT2, an important deacetylase, can deacetylate stable Ac-α-tubulin in microtubules, thus facilitating the differentiation of ependymal cells towards neurons [59].

By combining single-cell assays for transposase-accessible chromatin using sequencing (scATAC-seq) and single-cell RNA sequencing (scRNA-seq) of the injured spinal cord, the motifs for the canonical oligodendrocyte lineage transcription factors oligodendrocyte transcription factor 2 (OLIG2) and SRY-box transcription factor 10 (SOX10) were highly accessible not only in OPCs but also in ependymal cells, which shows that they unfold a latent gene expression program for oligodendrogenesis after injury. Furthermore, overexpression of the transcription factor OLIG2 in ependymal cells contributed to axon remyelination and improved functional recovery in Foxj1-Olig2-td Tomato mice [60].

#### Fibroblasts

Fibroblasts are the main producers of the matrix (including ECM) and constitute the basic framework of tissues and organs. In contrast, under normal conditions, fibroblast-like cells, of perivascular origin, are mostly related to the vasculature in the CNS and only contribute to the basement membrane [61]. SCI can induce a significant fibroblast response that produces matrix components. The resulting matrix components directly inhibit nerve regeneration and promote prolonged tissue remodeling through interactions with inflammatory cells. Spatially, these matrix components are separated by the surrounding reactive astrocytes, forming the fibrotic core of SCI scars. After an injury, PDGFRβ^+^ Glast^+^ vascular pericytes, namely type A pericytes, proliferate and cause fibrotic scar formation. When Glast1^+^ cell proliferation is prevented, it can lead to failure of wound closure, deterioration of lesion volume and reduction of matrix deposition [60], while a moderate reduction in pericyte-derived fibrosis can reduce scar pathology and achieve functional recovery [62]. Taken together, ECM plays regulatory roles in the developing and mature CNS. The components that stimulate active signaling within the ECM need further investigation for therapeutic use after SCI. Further research is needed to improve and optimize ECM-based strategies in order to induce anatomical repair and functional recovery following SCI.

### The role of glial scars

#### Beneficial effects on post-SCI repair

SCI promotes astrocyte migration and proliferation. Subsequently, reactive astrocytes fill the injured area and support the nerve tissue [61]. During the activation process, astrocytes enter the injury site together with other immune cells to isolate the injured area of the spinal cord and prevent the spread of tissue damage; in addition, they large release a number of cytokines, such as TNF-α, IL-1, IL-6, fibroblast growth factor (FGF) and nerve growth factor (NGF), to promote post-SCI repair. In 2016, [63] used the STAT3-cKO or TK + GCV mouse model to inhibit the proliferation of reactive astrocytes, which was found to ameliorate or prevent the formation of post-SCI glial scars. They found no glial scar formation in the acute phase and spontaneous regeneration in the descending corticospinal tract (CST), ascending sensory tract (AST) and serotonergic (5-HT) axons. In the chronic phase, axonal regeneration in these three types of nerve fibers into the injured area was not observed after the removal of glial scars formed by reactive astrocytes. In addition, AST axons were found to regenerate into the glial scar area upon concomitant stimulation with neurotrophin3 and brain-derived neurotrophic factor, and the glial scar was found to promote axon regeneration. However, inhibition of scar formation greatly reduced the regeneration of AST axons [61] as a consequence of the infiltration of blood-derived macrophages and fibrotic cells.

It is necessary to determine why infiltration blockage occurs and to elucidate the protective mechanisms. The role of microglia should be addressed in the prevention of parenchymal immunocyte infiltration. Bellver-Landete *et al*. took advantage of the Cx3c1-CreER mouse line with the CSF1R inhibitor PLX5622 to target microglia specifically following traumatic SCI [64]. They reported that extensive proliferation and accumulation of microglia around the damaged site occurred at 7 days, with a dense scar forming between the fibrotic scar and astrocytic scar. In contrast, the near-complete ablation of microglia by PLX5622 results in the reduction of IGF-1 with irregularly shaped and disorganized astroglial scar formation as well as secondary lesions filled with blood-derived inflammatory cells. This type of scene is horribly destructive for neuron regeneration. As a result, this was accompanied by a significant loss of neurons and NG2 cells within the damaged area, which further led to impairment of locomotor function. Accordingly, when M-CSF, the microglial proliferation factor, is delivered to the local site, local motor recovery is significantly improved as expected.

Above all, the glial scar is necessary to maintain tissue integrity and palliate further inflammatory reactions.

#### Detrimental effects on post-SCI repair

Several inhibitory substances in glial scars, such as Nogo-A, myelin-associated glycoprotein (MAG), NO, tenacin-R and CSPGs, are believed to inhibit axon regeneration. Silver and coworkers found that CSPGs secreted by reactive astrocytes in scar tissue can inhibit the growth of axons *in vitro* [65]. Currently, CSPGs are considered one of the main inhibitory factors that affect axonal regeneration. McMahon and coworkers found that digesting the CSPGs in the scar tissue at the injury site by intrathecal injection of chondroitinase ABC (ChABC) can effectively promote the regeneration of CST nerve axons and facilitate recovery of motor function to a certain extent [66]. In 2015, Silver and coworkers discovered that protein tyrosine phosphatase σ (PTPσ) in axons can interact with CSPGs and receptors in glial scars through Nogo receptors 1 and 3 to prevent axon growth. Subcutaneous injection of PTPσ mimic peptide was found to block the binding of PTPσ and CSPGs and restore axon regeneration, which effectively improved motor and urinary system functions in paralyzed mice after SCI [67]. In 2017, Barres and coworkers discovered that with the progression of SCI, the surrounding astrocytes were converted into scar-like astrocytes (also known as A1 type astrocytes) under the action of TNF-α, IL-1α and C1q secreted by microglia. These astrocytes lose their beneficial functions and can neither promote neuron survival, axon regeneration and synapse formation nor devour degraded myelin sheaths. Instead, the cells secrete harmful factors that induce the death of injured neurons and oligodendrocytes [68]. A1 eventually inhibits the regeneration of axons by forming a dense, chemical and mechanical barrier gel scar around the injured area. The mechanisms regulating the inhibitory transformation to A1 remain unclear, but evidence indicates that environmental factors, especially microglia-derived signals, are important.

#### Time-specific action of glial scars

A variety of cells are involved in scar tissue formation and the secretion of complex cytokines. There is no consensus on the exact role of glial scars in axonal regeneration. According to Prof. Silver, the contribution of glial scars to axonal regeneration is debatable [69]. In their study, CST regeneration was not observed in either the acute or chronic phases after specific removal of scars formed by reactive astrocytes; however, CST axon regeneration was not observed in the control group in which reactive astrocytes were not removed. Nonetheless, inhibition of scar formation during the acute phase impedes axon regeneration, which indicates that the removal of reactive astrocytes may induce changes in the local immune microenvironment. These findings suggest that reactive astrocytes are required for axon regeneration.

Dai and coworkers performed a proteomic analysis of scar tissues obtained from a rat model of complete SCI at 2 and 8 weeks after injury. At 2 weeks after SCI, the scar tissue contained a greater amount of bFGF, PDGF and vascular endothelial growth factor (VEGF), while at 8 weeks, the scar tissue contained a greater amount of CSPGs (CS-56); of note, CSPGs are not conducive to axon regeneration [70]. A possible explanation is that the effect of glial scars on nerve fiber regeneration changes dynamically over time and in different environments. In the early stage, glial scars are likely to promote axon regeneration by maintaining the stability of the internal environment, isolating the injured tissues and regulating the inflammatory response. However, obsolete glial scars may hinder and inhibit axon regeneration. On the one hand, glial scars and the secreted CSPGs can form a dense physical barrier that hinders axon regeneration. On the other hand, a variety of inhibitory signals can inhibit axon regeneration and remyelination, thereby impeding post-SCI functional restoration. Therefore, the identification of a suitable time window for SCI intervention that can help maximize the beneficial effects of glial scars is a key research imperative. Therefore, conditional ablation of different cell populations in the glial scar at a specific time is needed to better understand the effects of glia and the protein for axonal regeneration in time-specific action.

### Treatments

#### Surgical treatment

Surgical treatment in the acute phase aims to relieve spinal cord compression, prevent the death of neurons and glial cells and reduce the area of glial scars. Dai and coworkers observed obvious nerve fiber regeneration after surgical removal of old scar tissues at 8 weeks post-SCI. Their findings indicated that old glial scars affect axon regeneration after SCI [70]. In another study, nearly 60 patients with old SCI exhibited improved autonomic nerve function and extended sensory plane after removal of scar tissues lacking nerve conduction activity, in combination with collagen scaffold transplantation for nerve regeneration [71]. Therefore, the removal of old scar tissues can help improve sensory and motor functions.

#### High-dose methylprednisolone weakens glial scar formation

Methylprednisolone is a synthetic hormone with strong anti-inflammatory, immunosuppressive and anti-allergic properties. Administration of high-dose methylprednisolone in the early stage of SCI can reduce the release of inflammatory factors, alleviate post-traumatic spinal cord ischemia and minimize the death of spinal cord tissues and the area of glial scars.

#### Cell transplantation

To remove the local microenvironment of the glial scars and attenuate scar formation for axon regeneration, a cell transplantation method has been proposed [1]. In particular, the transplanted cells can provide nutritional support, neuroprotection and regulate inflammation after SCI, and can also form a bridge for nerve regeneration, thereby improving glial scars. Schwann cells [72], bone marrow mesenchymal stem cells, olfactory nerve sheath cells, fibroblasts expressing brain-derived neurotrophic factor and neurotrophin 3, and embryonic stem cells are commonly used for cell transplantation [73].

In a phase I clinical trial, transplantation of OPC1 (an oligodendrocyte progenitor cell line derived from human embryonic stem cells) into the cervical segment of SCI patients was found to reduce the cavity area and promote motor function recovery [74]. Fischer *et al*. first reported the recovery of bladder and motor functions after the transplantation of neuronal and glial restricted precursor cells [75]. Subsequently, injection of a lentivirus vector expressing neurotrophic factors into the injured site was found to promote the growth of axons up to 9 mm [76]. In subsequent years, Tuszynski and coworkers pioneered research on post-SCI neural stem cell transplantation. They found that in a rat model of completely transected SCI, the transplanted neural stem cells with co-expression of 10 neurotrophic factors not only survived for a long time, but also differentiated into mature neurons, and even formed synaptic connections with the host nerve fibers. The results showed significant improvement in the motor function of the rat forelimb [77]. They also demonstrated similar effects on functional recovery after transplantation of neural stem cells differentiated from healthy human induced pluripotent stem cells into a rat model of spinal cord hemisection [78]. Another study from Tuszynski’s laboratory also confirmed that CST axons can regenerate into and across the neural grafts in cervical and thoracic segments of SCI models [79].

In a landmark study using a rat cervical spinal cord impact model mimicking clinical SCI, the transplanted neural stem cells differentiated into neurons and glial cells, and the regenerated axons extended from the neurons toward the coracoid and caudal sides of the spinal cord and even formed synaptic contact with the host nerve fibers, leading to restoration of the forelimb motor function to some extent [80]. Tuszynski and coworkers further observed a similar recovery of forelimb motor function after transplantation of human spinal cord-derived neural stem cells into non-human primates [81]. In a recent study, Tuszynski’s group found that the regenerative transcriptome of the CST can be activated after SCI, and neural precursor cell transplantation can retain the characteristics of this regenerative transcriptome, rather than arousing new growth mechanisms to promote CST regeneration. They also found that the Huntington gene (Htt) is the central hub of the regenerative transcriptome and plays a key role in neuroplasticity after injury [82].

#### Cocktail therapy

In 2018, Sofroniew and coworkers reported the use of cocktail therapy comprising AAV virus overexpressing osteopontin, insulin growth factor, ciliary neurotrophic factor, FGF2, epidermal growth factor (EGF) and glial cell line-derived neurotrophic factor in a complete SCI model. They found that the axons emerging from the neurons regenerated beyond the glial scar and grew into gray matter, forming synaptic connections [83]. In particular, FGF2 and EGF augmented known axon growth-supportive substrates such as fibronectin, laminin and collagen in SCI [84,85]. However, FGF2 and EGF did not alter the expression of inhibitory CSPGs. FGF2 and EGF also increased astrocyte proliferation and cell density. Furthermore, integrin-dependent axon–substrate interactions with laminin, fibronectin or collagen are required for axon regrowth at the lesion site [83].

#### ChABC

ChABC is a lyase that degrades the chondroitin sulfate and dermatan sulfate chains of proteoglycan molecules. Most of the therapeutic effects of ChABC can be attributed to its ability to degrade the sugar chains of a class of proteoglycan molecules (CSPGs) [86]. This allows enzymes to degrade molecules that inhibit nerve regeneration and destroy structures rich in these molecules, namely the perineurium network (PNNs). ChABC has been widely used to eliminate the inhibitory activity of glial scars in different animal models. The pleiotropic effect of ChABC simultaneously targets multiple aspects of CNS damage, making it unique among the current technologies used to promote CNS repair and recovery. In addition, these activities do not overlap with most other therapies used to promote repair after SCI. Therefore, ChABC may be an important component of any combination therapy. It is combined with growth factors, transcription factors, cell transplantation, ion channels, agents that block remyelination inhibitors and agents that increase cAMP levels. In all cases, these effects were synergistic with those of ChABC [87]. Studies have shown that the combination of chondroitinase and cell transplantation is one of the most powerful combinations [87]. In fact, in two preclinical models of chronic SCI, combining ChABC+ rehabilitation without cell transplantation is sufficient to produce limited functional recovery [88,89]. In the case of acute SCI, it may be beneficial to add LLL [90] to reduce the subsequent inflammatory response. The use of scaffolds to deliver small therapeutic molecules, such as growth factors and cells, appears to be a promising strategy to enhance the growth of repaired and aligned axons. However, more work needs to be done in this area to determine the ideal materials for their construction. For the treatment of acute SCI, high-level and extensive enzyme delivery is associated with efficacy [91] and long-term delivery is associated with enhanced functional recovery [92]. Gene therapy, which also provides a method to strictly control the release level and time of the enzyme, can also be carried out. Once the treatment is completed, the delivery of the enzyme can be terminated. Gene therapy also leads to a large area of enzyme delivery to the spinal cord, which may be necessary for the effective treatment of human patients. In rats, continuous delivery of ChABC for 8 weeks was not associated with any adverse reactions [91,92].

Overall, blocking the pathway of CSPG in the injured area of glial scars has aroused wide therapeutic focus, together with encouraging but varying results. Specifically, conditional ablation of CSPGs from specific cell populations in the glial scar is needed to better understand the divergent and disparate roles of CSPGs during axon regeneration in order to improve promising therapeutic strategies.

#### X-Irradiation

X-Ray irradiation has a beneficial effect on SCI treatment. As early as 1963, X-ray radiation was found to have a significant effect on the spinal cord of newborn rats [93]. X-Ray irradiation can affect neurons and glial cells, although neurons are less sensitive to X-rays than are glial cells. A single-dose of X-ray irradiation with a D0 for X rays of 1.45 Gy does not damage nerve cells, especially oligodendrocytes, thereby promoting axon regeneration [94,95]. Kalderon and Fuks [96] found that X-rays can inhibit the death and degeneration of neurons and at least partially improve the recovery of motor function after spinal cord transection in rats. Results from Feng’s team showed that X-rays can improve the microenvironment of SCI and inhibit the formation of glial scars. They studied the effects of X-ray irradiation at different time points on the formation of glial scars after injury in rats and their effects on nerve function. The results confirmed that X-ray irradiation at a dose of 8 Gy inhibited the formation of glial scars at the injured site and reduced inflammation. The seventh day after injury may be the best time-window for local X-ray exposure [97].

### Problems and prospects

In a zebrafish SCI model, high expression of connective tissue growth factors in glial cells significantly promoted the bridging of defective nerves and even completely restored motor function [98]. Tuszynski and coworkers recently discovered that transplanted neural stem cells can differentiate into various subpopulations, forming the correct tissue morphology. In addition, motor and sensory nerve fibers were found to target the correct area, indicating successful restoration of the spinal cord circuit even in the absence of any endogenous targeting molecules [99,100]. These remarkable results of stem cell transplantation in zebrafish and rat models suggest that nerve regeneration is achievable. In a recent study, Fu and coworkers knocked down PTB (RNA-binding protein) in astrocytes to directly convert these cells into functional neurons; this is a potential novel therapeutic strategy for SCI [101]. Future studies should identify appropriate means of regulating and controlling glial scars, transforming glial scars into glial bridges and promoting axon regeneration into or beyond the glial scars, eventually restoring the function of the spinal cord circuit.

## Conclusions

The characteristics of glial scars are multifaceted for different types of injury and damage sites. Meanwhile, the scar consists of more than just the divergent glial constituents because there are multitudinous interactions between multiple different cell types (glial cells, mesenchymal-derived cells and immunocytes) with changes in intracellular components, signaling pathways and the extracellular environment. By these processes, cells within and around the glial scar are affected by and modulate each other. Altogether, the glial scar should be considered as a functional entity rather than simply be divided into different cell types. The environment of the scar, the secretion of glia, and the microenvironment, also known as the ECM, surround the glial cells. All of these factors contribute to mutual interactions between cells and the environment [102]. Consequently, the magnitude of inflammation is greatly affected. Spinal injury scars have both beneficial properties (blocking the spread of cellular damage and immunocyte infiltration) and detrimental properties (limiting new growth and tissue repair). This may be attributed to opposing functions of reactive glial cells that form the scar border. Therapeutic strategies [103] need to precisely target the detrimental aspects while preserving most of the beneficial components of the spinal injury scar. Increased mechanistic studies of the scar formation processes, which accurately subdivide each phenotype [104], would provide a deeper understanding of therapeutic strategies that may bring us closer to improving functional outcomes following SCI.

## Abbreviations

AST: ascending sensory tract; CSF1R:; ChABC: chondroitinase ABC; CNS:central nervous system; CreER: Cre estrogen receptor; CST: corticospinal tract; ECM: extracellular matrix proteins; EGF: epidermal growth factor; GFAP: glial fibrillary acidic protein; FGF: fibroblast growth factor; IL-1β: interleukin-1β; MAG: myelin-associated glycoprotein; NF-kB: nuclear factor-κB; NG2: neuron-glial antigen 2; NGF: nerve growth factor; OPC: oligodendrocyte progenitor cells; PDGFRα: platelet-derived growth factor receptor-α; RIP1: receptor-interacting protein 1; ROS: reactive oxygen species; SCI: spinal cord injury; TNF-α: tumor necrosis factor-α; TNFR1: tumor necrosis factor receptor 1; TRADD: TNF receptor-related death domain; TRAF2: TNF receptor-related factor 2; PTPσ: protein tyrosine phosphatase σ; TLR: toll-like receptors.

## Funding

This study was supported by the National Key Basic Research Program of China, No. 2017YFA0104701; The National Natural Science Foundation of China, No. 81801281, 81870975 and 81671230; and The Natural Science Foundation of Jiangsu Province (BK20202013).

## Authors’ contributions

Wrote the manuscript: YZ, SY, XH, XG, and SZ. All authors have read and approved the final manuscript.

## Conflicts of interest

The authors declare no conflict of interest.
